# Effect of a single injection of autologous conditioned serum (ACS) on tendon healing in equine naturally occurring tendinopathies

**DOI:** 10.1186/s13287-015-0115-0

**Published:** 2015-06-26

**Authors:** Florian Geburek, Maren Lietzau, Andreas Beineke, Karl Rohn, Peter M. Stadler

**Affiliations:** Equine Clinic, University of Veterinary Medicine Hannover, Foundation, Bünteweg 9, 30559 Hannover, Germany; Institute for Pathology, University of Veterinary Medicine Hannover, Foundation, Bünteweg 17, 30559 Hannover, Germany; Institute for Biometry, Epidemiology and Information Processing, University of Veterinary Medicine Hannover, Foundation, Bünteweg 2, 30559 Hannover, Germany

**Keywords:** Horse, Tendon, Ultrasonography, Biopsy, Histology, Collagen, Autologous conditioned serum, ACS, Irap

## Abstract

**Introduction:**

Autologous blood-derived biologicals, including autologous conditioned serum (ACS), are frequently used to treat tendinopathies in horses despite limited evidence for their efficacy. The purpose of this study was to describe the effect of a single intralesional injection of ACS in naturally occurring tendinopathies of the equine superficial digital flexor tendon (SDFT) on clinical, ultrasonographic, and histological parameters.

**Methods:**

Fifteen horses with 17 naturally occurring tendinopathies of forelimb SDFTs were examined clinically and ultrasonographically (day 0). Injured tendons were randomly assigned to the ACS-treated group (*n* = 10) receiving a single intralesional ACS injection or included as controls (*n* = 7) which were either untreated or injected with saline on day 1. All horses participated in a gradually increasing exercise programme and were re-examined nine times at regular intervals until day 190. Needle biopsies were taken from the SDFTs on days 0, 36 and 190 and examined histologically and for the expression of collagen types I and III by immunohistochemistry.

**Results:**

In ACS-treated limbs lameness decreased significantly until day 10 after treatment. Swelling (scores) of the SDFT region decreased within the ACS group between 50 and 78 days after treatment. Ultrasonographically, the percentage of the lesion in the tendon was significantly lower and the echogenicity of the lesion (total echo score) was significantly higher 78 and 106 days after intralesional ACS injection compared to controls. Histology revealed that, compared to controls, tenocyte nuclei were more spindle-shaped 36 days after ACS injection. Immunohistochemistry showed that collagen type I expression significantly increased between days 36 and 190 after ACS injection.

**Conclusions:**

Single intralesional ACS injection of equine SDFTs with clinical signs of acute tendinopathy contributes to an early significant reduction of lameness and leads to temporary improvement of ultrasonographic parameters of repair tissue. Intralesional ACS treatment might decrease proliferation of tenocytes 5 weeks after treatment and increase their differentiation as demonstrated by elevated collagen type I expression in the remodelling phase. Potential enhancement of these effects by repeated injections should be tested in future controlled clinical investigations.

## Introduction

Tendinopathy of the superficial digital flexor tendon (SDFT) is a common injury in Thoroughbred racehorses and other horse breeds and is regarded as a career-limiting disease with a high recurrence rate [1]. Numerous treatment modalities have shown limited success in improving tendon repair [2]. Regenerative therapy aims to restore structure and function after application of biocompatible materials, cells, and bioactive molecules [3, 4]. There is growing knowledge about the clinical effects of potentially regenerative substrates, e.g. mesenchymal stem cells (MSCs) [5, 6] and autologous blood products such as platelet rich plasma [7, 8] on equine tendinopathies. To date, however, ideal treatment strategies for naturally occurring tendinopathies have not been established [1, 2].

Autologous conditioned serum (ACS; synonyms irap®, Orthokine®, Orthogen, Düsseldorf, Germany) is used for intralesional treatment of tendinopathy in horses but, to the best of our knowledge, its clinical effect is only documented anecdotally [8–10]. ACS is prepared by exposing whole blood samples to glass beads, which has been shown to stimulate the secretion of anti-inflammatory cytokines, including interleukin (IL)-4 and IL-10 and IL-1 receptor antagonist (IL-1Ra) in humans [11]. A recent investigation has shown that ACS from equine blood also contains high levels of IL-1Ra and IL-10 [12]. Equine studies have focused on the IL-1Ra-mediated anti-inflammatory effects of ACS [13]; however, in tendon healing, the high concentrations of growth factors such as insulin-like growth factor-1 (IGF-1) and transforming growth factor-beta (TGF-β) may be equally or more important [14–16]. Blood samples from different horses and the use of different kits for the preparation of ACS may lead to differences in the cytokine and growth factor concentration in vitro [12, 17]. However, the relevance of these differences for the clinical effect is unknown.

ACS was originally described to improve muscle regeneration in a murine muscle contusion model [18] and to exhibit anti-inflammatory effects in an experimental model of carpal osteoarthritis in horses [13] and in a placebo-controlled clinical trial in humans with knee osteoarthritis [19].

The rationale for the use of ACS to treat equine tendinopathies is based on several findings: 1) It was shown in an experimental study that the expression of IL-1β (and matrix metalloproteinase-13) is upregulated following overstrain injury of rat tendons, demonstrating that these molecules are important mediators in the pathogenesis of tendinopathy [15, 20]. 2) IL-1Ra protein and heterologous conditioned serum prepared with the irap® kit reduced the production of prostaglandin E_2_ by stimulated cells derived from macroscopically normal SDFTs in vitro [21]. 3) Growth factors concentrated in ACS, e.g. IGF-1 and TGF-β, have the potential to attract resident precursor cells, e.g. MSCs and tenoblasts, and to increase cell proliferation during tendon healing [14, 15, 17]. Rat Achilles tendons exposed to ACS in an experimental study showed an enhanced expression of the Col1A1 gene, which led to an increased secretion of type I collagen and accelerated recovery of tendon stiffness and improved histologic maturity of the repair tissue [22]. It was shown in another rodent Achilles tendon transection model that ACS generally increases the expression of basic fibroblast growth factor (bFGF), bone morphogenetic protein-12 and TGF-β1 [16], representing growth factors important for the process of tendon regeneration [15].

The process of tendon healing is mainly divided into three phases which merge into each other. The acute inflammatory phase (<10–14 days) is characterized by phagocytosis and demarcation of injured tendon tissue. A fibroproliferative callus is formed during the proliferative phase (4–45 days), while collagen fibrils are organised into tendon bundles during the remodelling or maturation phase (45–120 days; <3 months) [1, 23].

The aim of the present study was to support the hypothesis that a single intralesional ACS injection into SDFT lesions 1) has a clinically detectable anti-inflammatory effect, 2) leads to improved B-mode ultrasonographic parameters and 3) improves the organization of repair tissue.

## Materials and methods

Inclusion criteria for client-owned adult horses was a history of acute uni- or bilateral SDFT tendinopathy (tendon disorder) without cutaneous injury but with clinical signs of inflammation being reported to be present for up to 14 days prior to the presentation at the Equine Clinic of the University of Veterinary Medicine, Hannover, Foundation, or to collaborating veterinarians. Horses were only included if the clients agreed to the study design and tendons had not received intralesional injections before. Injured limbs were randomly assigned to the group treated with ACS (*n* = 10) or to controls (*n* = 7). The study was carried out between 2009 and 2012 and approved by the animal welfare officer of the University of Veterinary Medicine Hannover, Foundation, Germany, and the ethics committee of the responsible German federal state authority in accordance with the German Animal Welfare Law (Lower Saxony State Office for Consumer Protection and Food Safety, Az. 33.9-42502-05-09A652).

### Clinical examination

All horses were examined clinically on the day of first presentation (day 0). This examination included visual assessment of lameness (5 grade score) [24] and signs of inflammation which were scored semiquantitatively by palpation (skin surface temperature in the palmar metacarpal region and sensitivity of the SDFT to palpation: 0 = no abnormality, 1 = mild abnormality, 2 = moderate abnormality, and 3 = severe abnormality; swelling of the SDFT was determined by palpation as an increase in diameter relative to normal tendon: 0 = no increase, 1 = increase by factor 1.5; 2 = increase by factor 1.5 to 2; increase by more than factor 2 [25]).

### B-mode ultrasonography

All injured tendons were examined with B-mode ultrasound on the day of first presentation (day 0) in a transverse and longitudinal fashion with a linear 5–7.5 MHz linear scanner (Logiq e, GE Healthcare, Wauwatosa, WI, USA), according to the seven zone designations as described previously [26, 27]. Images were stored digitally and analysed according to the following parameters to determine the degree- and time-related changes of the lesions: maximal injury zone (MIZ), type of lesion determined on transverse images in the MIZ (core lesion = centrally located, focal hypo-/anechoic region; marginal lesion = peripherally located, focal hypo-/anechoic region; diffuse lesion = homogenous or heterogenous changes in echogenicity of the whole/most parts of the cross sectional area), summarized cross-sectional areas of the tendon (total cross-sectional area, T-CSA), summarized cross-sectional areas of the lesion (total lesion cross-sectional area, TL-CSA), and percentage of the lesion in the tendon [percent total lesion, %T-lesion = (TL-CSA / T-CSA) × 100]. Echogenicity and fibre alignment were graded semiquantitatively at each zone and the scores for all levels were summarized (total echo score, TES; total fibre alignment score, T-FAS). Echogenicity was assigned to 0 (normoechoic), 1 (hypoechoic), 2 (mixed echogenicity), and 3 (anechoic) [27, 28], and fibre alignment was graded according to the estimated percentage of parallel fibres in the lesion: 0 (>75 %), 1 (50–74 %), 2 (25–49 %), and 3 (<25 %) [27, 28]. Analyses of ultrasonograms were performed by one examiner (ML) blinded to the individual treatment modality.

### Intralesional treatment, follow-up examinations and controlled exercise

Ten millilitres of autologous blood were collected by a single venipuncture of one jugular vein into an irap®-10 syringe system (Orthogen, Düsseldorf, Germany). Blood samples were incubated at 37 °C (range 6–9 hours). After centrifugation at 4,000 rotations per minute for 10 minutes (centrifuge: Universal 320, rotor: no. 1624, Hettich, Tuttlingen, Germany), serum was aseptically aspirated from the syringe and passed through a 0.22 μm syringe-driven filter unit (Millex-MP, Millipore Corporation, Carrigtwohill, Co. Cork, Ireland). Depending on the size of the lesion as determined ultrasonographically (TL-CSA), tendons allocated to the ACS group received a single intralesional injection of 1–3 ml through a 22G needle (diameter 0.7 mm, length 30 mm) into the SDFT defect (day 1). Control tendons either received a single injection of a placebo, i.e. 1–3 ml saline through a 22G needle (diameter 0.7 mm, length 30 mm) or were untreated in case the owner declined an intralesional application of saline. Horses were sedated for the intralesional injections with detomidine (0.01–0.03 mg/kg intravenously) and butorphanol (0.04–0.05 mg/kg intravenously), and the medial and lateral palmar nerves were anaesthetized 2 cm distal to the carpometacarpal joints with 2 ml of a 2 % mepivacaine solution. After aseptic preparation of the skin, superficial digital tendon lesions were injected under sonographic guidance at a single site from the lateral aspect of the tendon perpendicularly to its long axis directly into the most hypoechoic areas, i.e. the MIZ while the limb was weight-bearing. All horses participated in a gradually increasing exercise programme as described previously [7]. The programme started the first day after the reported onset of SDFT tendinopathy. From week 25 to 27 horses were exercised for 25 minutes at a walk and for 15 minutes at a trot.

Horses were re-examined clinically and ultrasonographically at regular intervals for 27 weeks on days 11, 22, 36, 50, 78, 106, 134, 162, and 190. Thereafter horse owners were advised to gradually increase exercise on an individual basis until the previous level of performance was reached. Data concerning signs of acute tendon injury, the level of performance horses reached and the discipline they were used for were obtained by telephone inquiry with horse owners or trainers until the preparation of the manuscript.

### Needle biopsies and histologic examinations

On days 0, 36 and 190, one needle biopsy was taken aseptically from each SDFT at its MIZ with a 20G automated biopsy needle (Biopsiepistole PlusSpeed™, Peter Pflugbeil GmbH, Zorneding, Germany), with the needle entering the MIZ of the SDFT from distal at a 45° angle while the carpus was flexed approximately 90° and the metacarpophalangeal joint was moderately extended [29]. Pain reaction and intensity of bleeding from the biopsy site were evaluated using an established score [29]. The MIZ was recorded as distance from the accessory carpal bone (cm) so that repeat biopsies were taken from the same anatomic area as the day 0 biopsy while avoiding previous biopsy sites. Limbs were protected with a distal limb bandage for 2 days after taking needle biopsies and after intralesional injections. All needle biopsies were fixed in 10 % formalin, paraffin-embedded, sectioned at a thickness of 1–2 μm, mounted on microscope slides, and stained with haematoxylin and eosin. A single histological slide of each biopsy was examined histologically according to a score described previously [7, 30]; findings were graded using a semiquantitative four-point scale (0 = normal appearance, 1 = slightly abnormal, 2 = moderately abnormal, and 3 = markedly abnormal) considering the following parameters: fibre structure (0 = linear, no interruption; 3 = short with early truncuation), fibre alignment (0 = regularly ordered; 3 = no pattern identified), morphology of tenocyte nuclei (0 = flat; 3 = round), variations in cell density (0 = uniform; 3 = high regional variation), and vascularisation (0 = absent; 3 = high). Histological sections were independently scored by two observers blinded to horse and treatment modality (FG and ML). In total, five high power fields (40× magnification) per section were examined and scored. Mean averages of score values determined by each observer were calculated for each parameter (see above) before score values of both examiners were averaged.

Immunohistochemical analysis of paraffin-embedded tissue sections was used to determine the formation of collagen type I and collagen type III. A commercially available mouse-anti-bovine antibody (NB600-450 anti-COL 1A1, Novus Biologicals, Littleton, CO, USA) and a rabbit-anti-bovine antibody (CL197P anti collagen type III alpha 1 chain, Acris Antibodies GmbH, Herford, Germany) were applied as primary antibodies against collagen type I and collagen type III, respectively. Secondary biotinylated antibodies were obtained from relevant species allowing binding to the primary antibody. Colour production from the chromogen diaminobenzidine tetrachloride was catalysed by streptavidin-conjugated peroxidase (avidin-biotin-complex-method) [31]. Finally, the sections were counterstained with haematoxylin. Immunohistochemical cross-reactivity of antibodies with uninjured equine tendon tissue was tested prior to analysis of the needle biopsies. Positive control tissues included bovine aorta and bovine tendon for collagen type I antigen-specific antibodies and bovine skin for collagen type III antigen-specific antibodies. In negative control sections, primary antibodies were replaced by appropriately diluted Balb/c mouse ascites and rabbit serum, respectively.

Photomicrographs were taken from all immunostained slides (Color View II, 3.3 Megapixel CCD, Soft Imaging System GmbH, Münster, Germany). Quantitative morphometric analysis of the immunoreaction was achieved by determination of the immunostained area using image analysis software (analySIS® 3.1, Soft Imaging System GmbH, Münster, Germany). A threshold for a positive signal was defined and the percentage of positively immunostained area in the tissue section as a whole was calculated [32, 33].

### Statistical analysis

Analysis of data was performed using SAS™ Version 9.3 (SAS Institute, Cary, NC, USA). The level of significance was set at *p* < 0.05. All values in the graphs are expressed as arithmetic mean values with standard error ($\overline{\mathrm{X}}$ ± SEM). The assumption of normality was tested using the Kolmogorov-Smirnov test and visual assessment of qq-plots of model residuals. In the case of rejection of normal distribution, distribution-free nonparametric methods were applied. Fisher’s exact test was applied to test the differences between groups on each examination day with regard to the parameters of degree of lameness, swelling and skin surface temperature. To compare not-normally distributed parameters within a group between examination days, the permutation test for nonparametric analysis of repeated measurements with the Šidák post hoc test for multiple pairwise comparisons was used. The influence of groups and time points on ultrasonongraphic parameters (T-CSA, TL-CSA, %T-lesion, TES and T-FAS), histology scores and percentages of positively immunostained areas were tested using a two-way analysis of variance for independent samples (groups) and repeated measurements (dependent time points, biopsies), followed by the Tukey post hoc test for multiple pairwise comparisons. The intraclass correlation coefficient was calculated by analysis of variance components to test inter-observer repeatability of histological scores.

## Results

### Description and history of horses, intralesional injections

Seventeen limbs of 15 horses between 2 and 19 years old (mean 8.46 years old) met the inclusion criteria. Ten of the limbs were included in the ACS-treated group (ACS group) and seven served as controls. Limbs allocated to the ACS group belonged to five Warmbloods (50 %), four Thoroughbreds (40 %) and one Arabian (10 %). The limbs of five Warmbloods (71.42 %), one Thoroughbred (14.28 %) and one Half-blood (14.28 %) were included as controls. Of these, one Thoroughbred and one Warmblood with bilateral SDFT lesions served for both groups (Table 1). The horses’ history (i.e. high-speed exercise, increased age >12 years) was suggestive of tendinopathy to be strain-induced in at least 6 of 10 SDFTs (60 %) allocated to the ACS group and in at least 4 of 7 (57 %) control SDFTs. Two tendons had a definitive history of blunt external trauma. One was included in the ACS group and one served as control.

**Table 1:** Description, clinical history, diagnostic data, and treatment of 15 horses with 17 SDFT lesions

Horse number	Breed	Age (years)	Gender	For which purpose used	Reported duration of SDFT tendinopathy until initial examination (days)	Reported initiating event	Limb affected	Maximal injury zone	Lesion type	Treatment
**ACS group**										
2241/09	Thoroughbred	2	S	Racing	2	Training	RF	2b	Diffuse	ACS
2240/09	Thoroughbred	3	S	Racing	2–3	Training	RF	2b	Core	ACS
2489/09^a^	Thoroughbred	4	M	Racing	7	Racing	RF	1b	Core	ACS
2539/09	Warmblood	3	S	Dressage	14	Blunt trauma	RF	2b	Marginal	ACS
1672/10	Arabian	17	G	Pleasure	14	Running free	RF	1b	Core	ACS
6264/10^b^	Warmblood	8	G	Dressage	9	Unknown	RF	3a	Marginal	ACS
6335/10	Warmblood	10	G	Pleasure	10	Unknown	LF	2b	Diffuse	ACS
4793/10	Thoroughbred	3	S	Racing	7	Training	LF	2b	Core	ACS
6263/10	Warmblood	20	M	Pleasure	14	Stumbling at cross country ride	RF	1b	Core	ACS
2378/10	Warmblood	11	M	Pleasure	13	At ride	LF	2b	Diffuse	ACS
		Mean 8.1								
**Controls**										
2489/09^a^	Thoroughbred	4	M	Racing	7	Racing	LF	1b	Diffuse	No
6264/10^b^	Warmblood	8	G	Dressage	9	Unknown	LF	2a	Core	Saline
6111/10	Warmblood	5	M	Jumping	14	Kicking himself over the jump	RF	1b	Marginal	No
6265/10	Warmblood	5	M	Pleasure	7	Unknown	RF	1b	Marginal	Saline
6383/11	Warmblood	18	M	Pleasure	10	At cross country ride	LF	2a	Marginal	No
5461/11	Warmblood	14	G	Police horse	4	At gallop on beach	LF	2b	Diffuse	No
6384/11	Half-blood	8	G	Eventing	1	After eventing competition	LF	2a	Core	No
		Mean 8.86								

^a, b^Horses had bilateral SDFT lesions and served for the ACS group and as control. *ACS* autologous conditioned serum (treated with single intralesional injection of autologous conditioned serum); *G* gelding, *LF* left front limb, *M* mare, *RF* right front limb, *S* stallion, *Saline* treated with single intralesional saline injection, *SDFT* superficial digital flexor tendon

In total ten SDFTs were injected with ACS. Five control SDFTs were not treated and two control tendons received a single intralesional injection of saline.

### Lameness

**Fig. 1:**
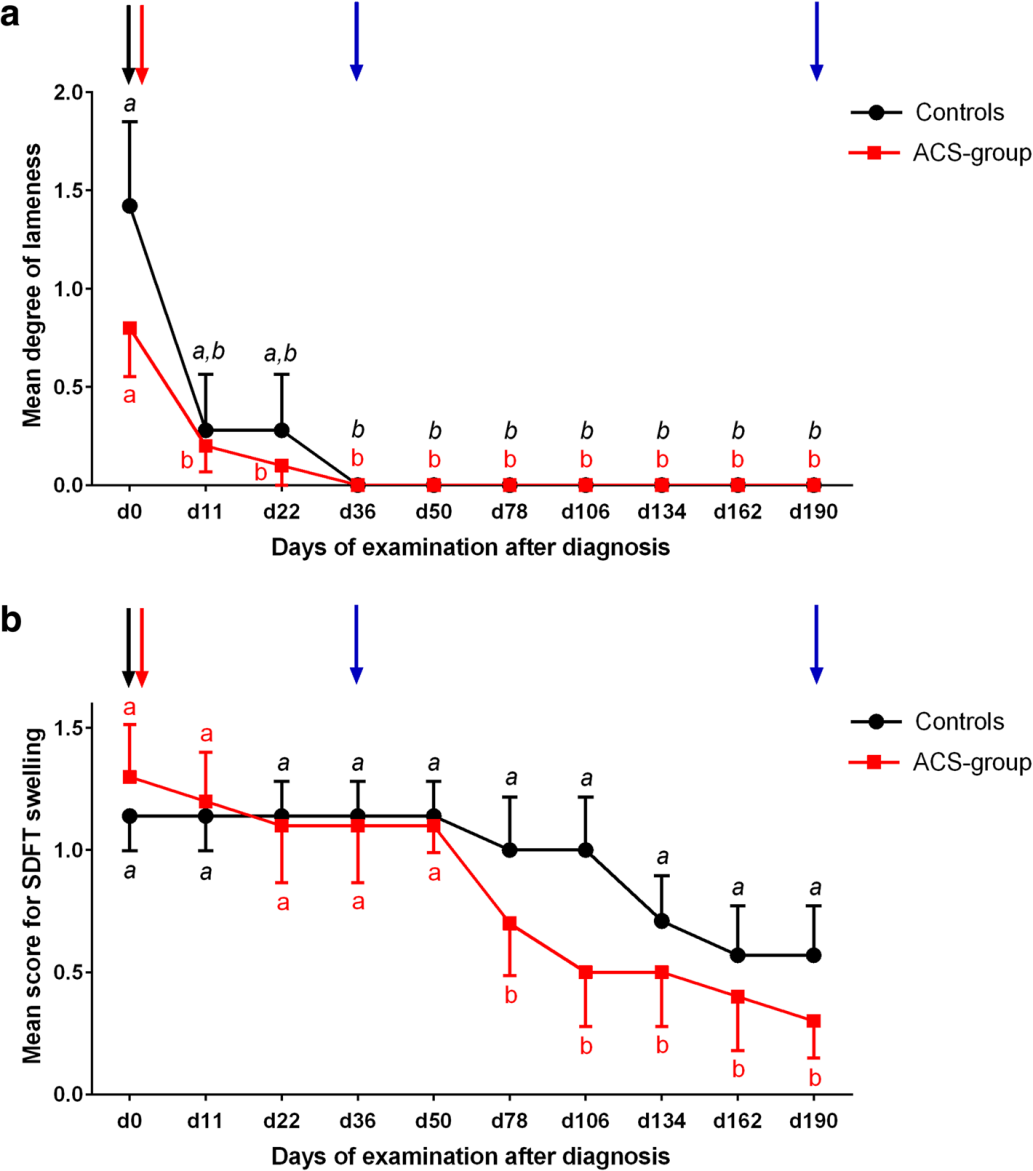
Degree of lameness and palpable swelling of tendons. **a** Degree of lameness of control limbs and those treated with autologous conditioned serum (ACS) over time. **b** Scores for palpable swelling of ACS-treated and control superficial digital flexor tendons (SDFTs) over time. Mean ± SE. Different letters (ACS *normal*, and control *italic*) indicate significant differences (*p* < 0.05) within treatment group. ACS-group, *n* = 10 limbs (SDFTs treated with a single injection of ACS); controls, *n* = 7 limbs (SDFTs treated with a single injection of control substance or left untreated). *Black arrow* day (d)0 – diagnosis, first tendon biopsy; *red arrow* d1 – intralesional injection of ACS/control substance; *blue arrows* d36/d190 – second/third tendon biopsy

On day 0, the mean degree of lameness was 0.8 in the ACS group. In control limbs, the mean degree of lameness was 1.42 on day 0. One of the two horses with bilateral tendinopathy that served for the ACS group and as a control was not lame. The second one showed a unilateral front limb lameness (SDFT in the lame limb was treated with ACS). The mean degree of lameness did not differ between groups on any day of examination (*p* > 0.05). Regardless of treatment modality all horses became sound by day 36. Compared to day 0, lameness decreased significantly by day 11 (*p* = 0.046) within the ACS group and it decreased significantly by day 36 (*p* = 0.021) in limbs serving as controls (Fig. 1a).

### Long-term follow-up

Recurrence of tendon injury was not reported in any horse after 2 to 4 years post-diagnosis. Of the eight horses with SDFTs being allocated to the ACS group, five horses (63 %), among these three racehorses, returned to their previous or a higher performance level, two horses (25 %) died of reasons unrelated to tendinopathy, and one horse (12 %) was retired due to osteoarthritis of interphalangeal joints after the observation period. One of the horses which was included in the ACS group and served as a control did not resume training and was retired as a broodmare; the second one performed as a dressage horse. Of the five horses with tendons serving only as control, four individuals (80 %) performed in their discipline at the previous or a higher level, and one horse (20 %) was lost to follow-up.

### Signs of inflammation

No statistically significant differences between groups were observed during the entire observation period, including day 0, with regard to scores for swelling, skin surface temperature and sensitivity to palpation. Swelling scores of the SDFT region decreased significantly in the ACS group (*p* = 0.005) between day 50 (mean score 1.1) and day 78 (mean score 0.7) and remained reduced until the end of the observation period (Fig. 1b). In controls, swelling scores did not decrease significantly during 27 weeks. Skin surface temperature and sensitivity to palpation scores decreased up to day 22 within both groups and remained at a low level until day 190.

### B-mode ultrasonography

The horses included presented with core lesions (seven limbs), marginal lesions (five limbs) or diffuse lesions (five limbs) of the SDFT (Table 1). The MIZ of most lesions was located in zone 2b (41.17 % of limbs), followed by zone 1b (35.29 % of limbs), zone 2a (17.64 % of limbs) and zone 3a (5.88 % of limbs).

**Fig. 2:**
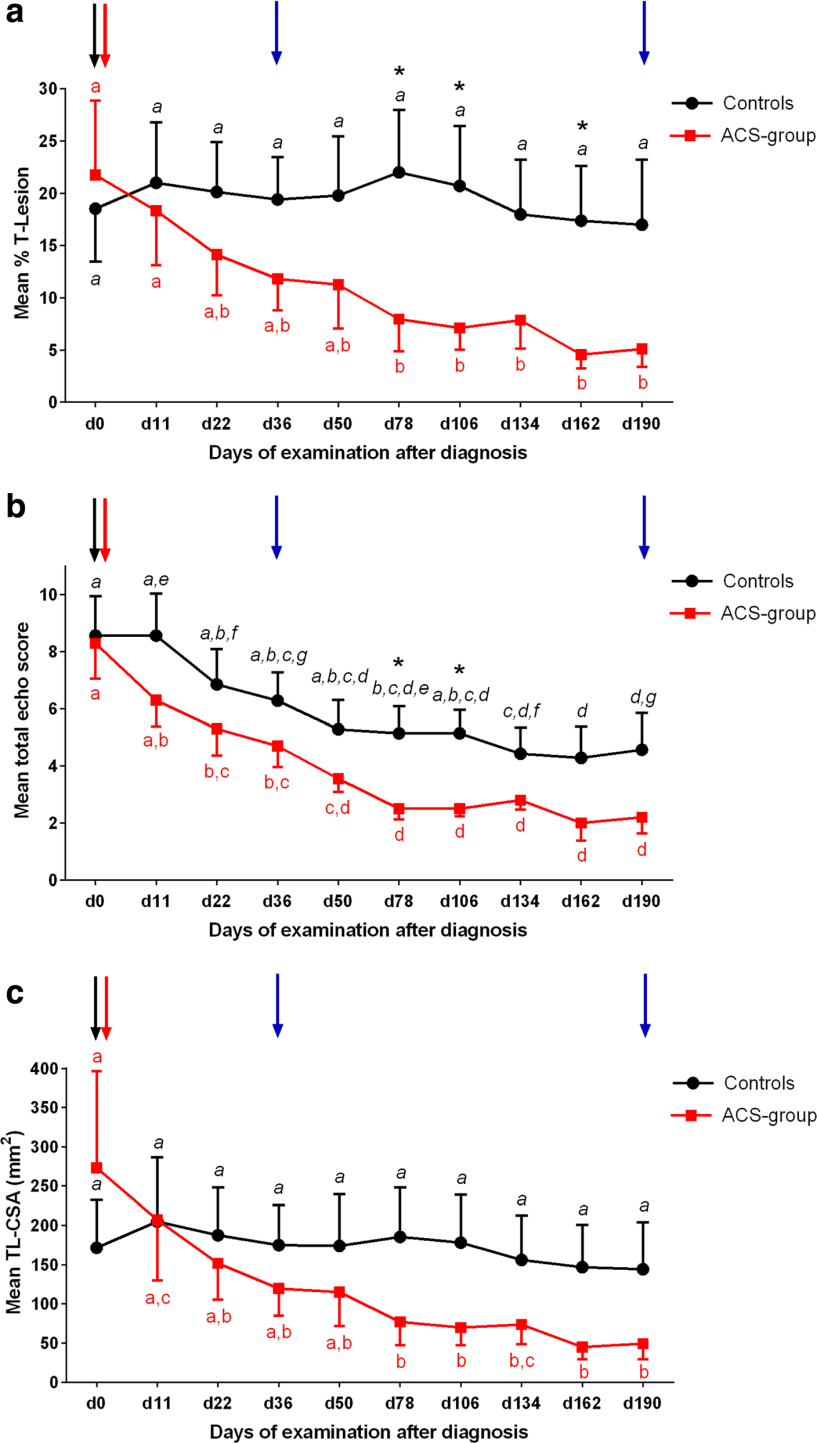
Ultrasonographic measurements. **a** Percent total lesion (%T-Lesion) of autologous conditioned serum (ACS)-treated and control superficial digital flexor tendons (SDFTs) over time. **b** Total echo scores of ACS-treated and control SDFTs over time. **c** Total lesion cross-sectional area (TL-CSA) of ACS-treated and control SDFTs over time. Mean ± SE. *p < 0.05, between groups. Different letters (ACS *normal*, and control *italic*) indicate significant differences (*p* < 0.05) within treatment group. ACS-group, *n* = 10 limbs (SDFTs treated with a single injection of ACS); controls, *n* = 7 limbs (SDFTs treated with a single injection of control substance or left untreated). *Black arrow* day (d)0 – diagnosis, first tendon biopsy; *red arrow* d1 – intralesional injection of ACS/control substance; *blue arrows* d36/d190 – second/third tendon biopsy

The mean %T-lesion was 21.73 ± 7.16 in the ACS group and 18.51 ± 5.07 in controls on day 0. This parameter was significantly lower (*p* < 0.05) in the ACS group than in controls on days 78, 106 and 162 (Fig. 2a). TES were significantly lower in the ACS group versus controls on days 78 and 106 (Fig. 2b). There was no difference in T-CSA, TL-CSA (Fig. 2c) or T-FAS between the groups at any time point.

Mean %T-lesion showed a continuous decrease over time within the ACS group which was significant (*p* = 0.02) for the first time on day 78 compared to day 0. After this period, this parameter remained below the 10 % range. In control tendons, mean %T-lesion continuously remained on a similar elevated level (*p* > 0.05) throughout the entire observation period (Fig. 2a).

Compared to day 0, the mean TES decreased significantly on day 22 and again between days 22 and 78 in the ACS group (*p* < 0.05), while in controls compared to day 0 the TES decreased significantly (*p* < 0.05) the first time on day 78 (Fig. 2b).

Mean T-CSA did not change significantly throughout the observation period in either group. Regarding the progression of TL-CSA within the ACS group over time, it was significantly lower (*p* < 0.05) from day 78 onwards until the end of the examination period compared to levels on day 0, while values from control tendons remained on a similar level from day 0 to 190 (Fig. 2c). Mean T-FAS decreased significantly between day 0 and day 78 (*p* = 0.023) within the ACS group; in controls this parameter decreased significantly on day 134 compared to day 0 (*p* = 0.04).

### Needle biopsies and histology

A total of 51 needle biopsies were taken. Pain reaction was mild in 78.43 % of the procedures, mild to moderate in 17.64 % and moderate in 3.92 % of the cases. A moderate to severe or severe pain reaction was not observed. No bleeding was observed after taking biopsies in 25.49 % of the cases, mild bleeding occurred in 58.82 % and moderate bleeding in 15.68 % of cases. Severe bleeding was not observed.

Of 51 biopsies, 47 were available for tendon histology [7, 30]. Four biopsies from severely oedematous lesions were not evaluated due to limited tissue content. The intra-class correlation for inter-observer repeatability was 0.72 for fibre structure, 0.88 for fibre alignment, 0.84 for nuclei morphology and 0.92 for variations in cell density.

**Fig. 3:**
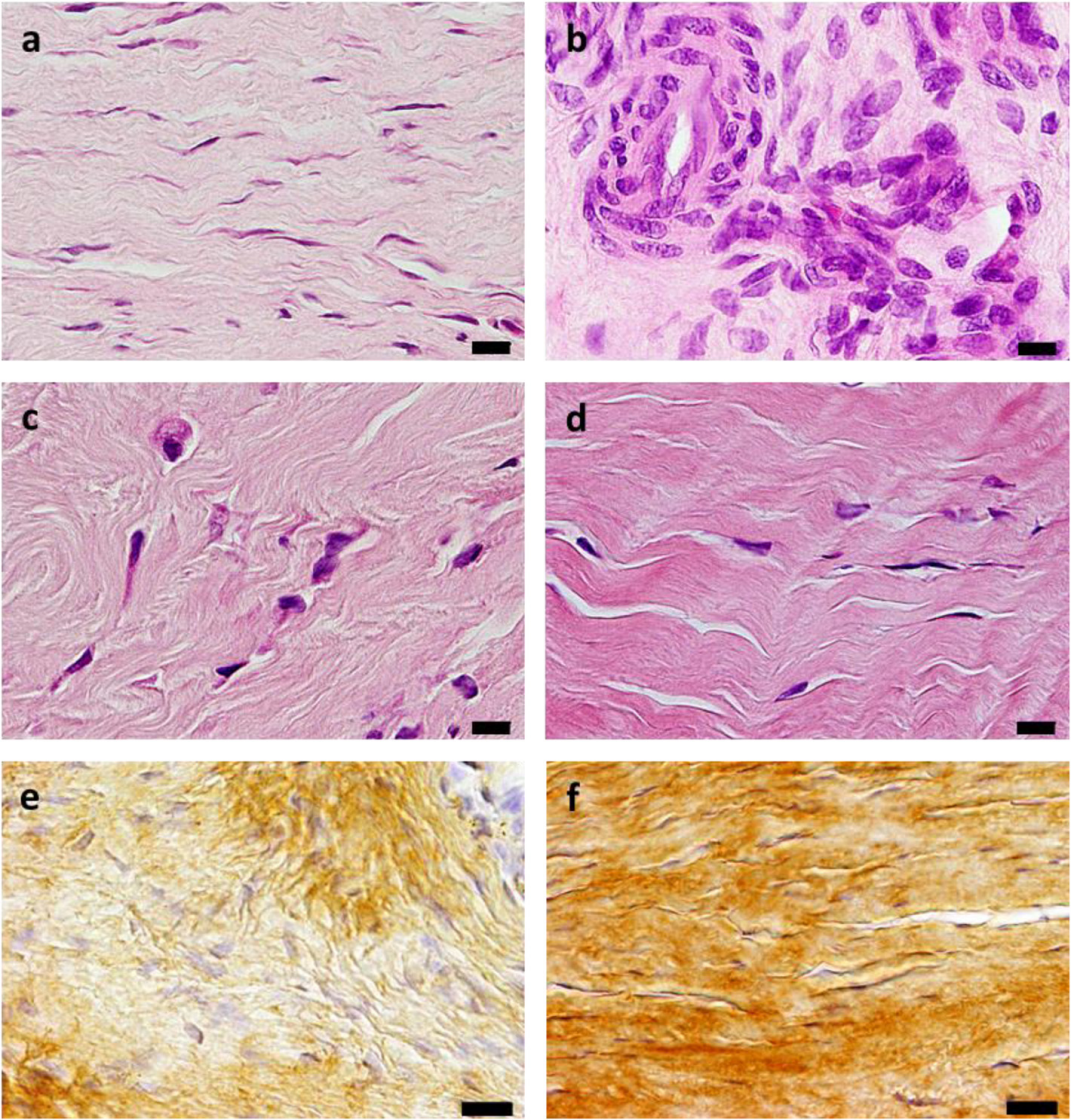
Longitudinal sections of tendon biopsies from superficial digital flexor tendons with tendinopathy. **a–d** Histopathological specimens stained with haematoxylin & eosin using a 40× objective. Tendons of horses on day 36 after intralesional treatment with autologous conditioned serum (ACS) (horse no. 4793/10; **a**) and no treatment (control tendon, horse no. 6384/11; **b**). The number of round cell nuclei was higher in control tendons than in ACS-treated tendons 36 days after treatment. Scale bars = 10 μm. Tendon of horse no. 2241/09 1 day before (day 0, **c**) and 190 days after (**d**) intralesional treatment with ACS. Alignment of collagen fibres improved significantly between day 0 and day 190 after ACS treatment. Scale bars = 10 μm. Tendon of horse no. 2240/09 36 days (**e**) and 190 days (**f**) after intralesional treatment with ACS. Immunohistochemistry revealed a significant increase of collagen type I expression between day 36 and day 190 after ACS treatment. Scale bars = 20 μm

**Fig. 4:**
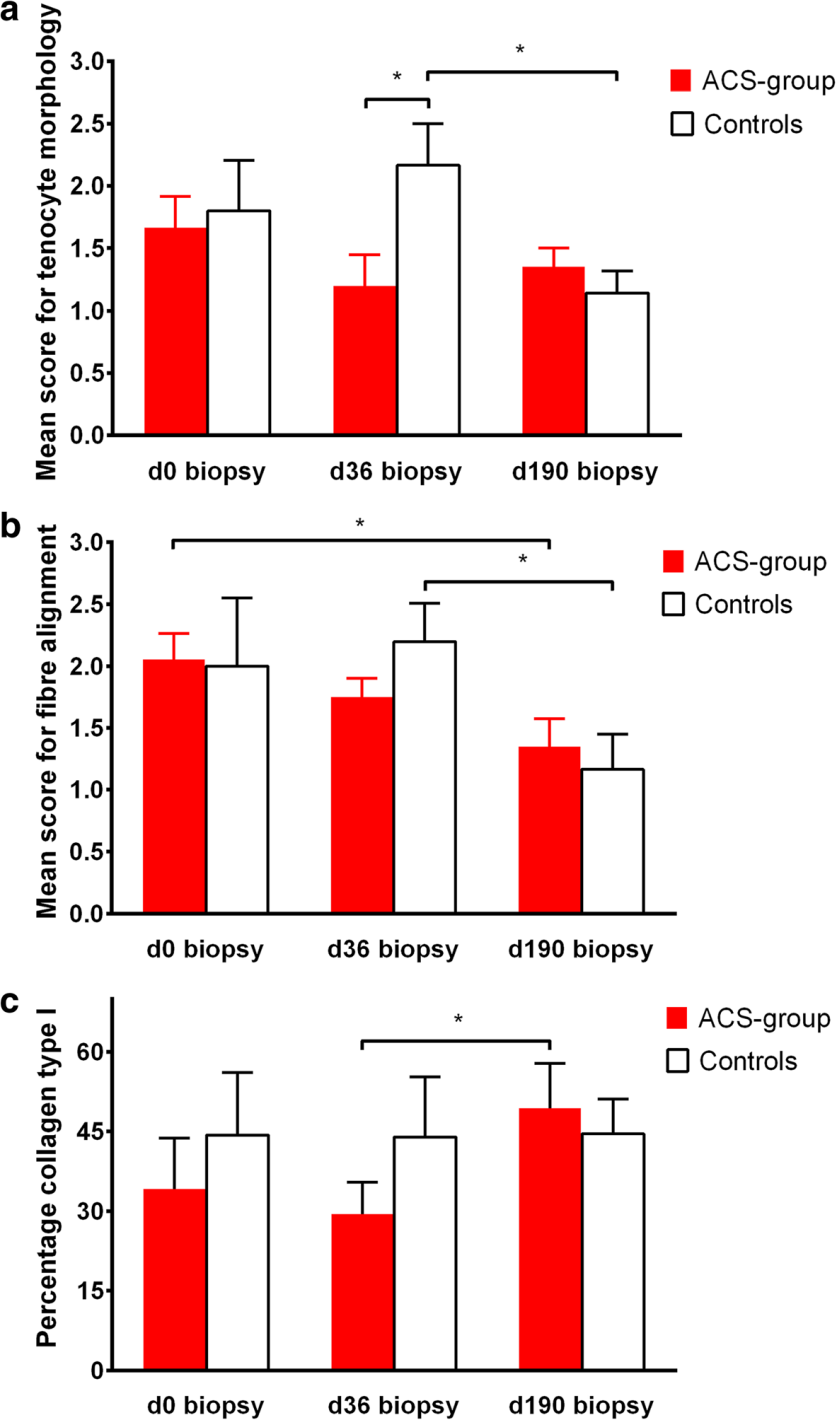
Histologic scores and collagen type I content of superficial digital flexor tendons. **a** Histologic scores for morphology of tenocyte nuclei in tendon biopsies taken from autologous conditioned serum (ACS)-treated versus control superficial digital flexor tendons (SDFTs) at different time points during the examination period of 190 days. **b** Histologic scores for fibre alignment in tendon biopsies taken from ACS-treated versus control SDFTs at different time points during the examination period of 190 days. **c** Percentage of collagen type I content determined immunohistochemically in tendon biopsies taken from ACS-treated versus control SDFTs at different time points during the examination period of 190 days. Day (d)0 = day the diagnosis was made; d36/d190 = 36/190 days after tendinopathy was diagnosed. *p < 0.05. ACS group, *n* = 10 limbs (SDFTs treated with a single injection of ACS); controls, *n* = 7 limbs (SDFTs treated with a single injection of control substance or left untreated)

Scores for tenocyte nuclei morphology were significantly lower (i.e. cell nuclei more flattened; *p* = 0.01) in the ACS group (Fig. 3a) than in controls (Fig. 3b) on day 36 (Fig. 4a). Scores for cell density showed a tendency to be lower (i.e. more uniform) in the ACS group than in controls on day 36 (*p* = 0.052). Scores for fibre structure, fibre alignment (Fig. 4b), vascularisation, and subscores for structural integrity and metabolic activity did not show differences between the ACS group and controls at any time point.

With regard to development of fibre alignment during the healing of tendons treated with ACS, scores for this parameter were significantly lower (i.e. fibres were more regularly ordered; *p* = 0.04) in biopsies taken at the end of the observation period (day 190; Fig. 3d, Fig. 4b) than in those taken on day 0 (Fig. 3c, Fig. 4b). In control tendons, scores for fibre alignment (Fig. 4b), scores for morphology of tenocyte nuclei (Fig. 4a) and scores for cell density decreased significantly (i.e. tissue morphology improved; *p* < 0.05) between day 36 and day 190 biopsies.

There were no differences between the ACS group and controls with regard to collagen type I and collagen type III expression in biopsies taken on days 0, 36 and 190. Within the ACS group, the collagen type I content increased significantly (*p* = 0.03) between the biopsies taken on day 36 (Fig. 3e) and day 190 (Fig. 3f), while it remained at the same level (*p* > 0.05) in controls (Fig. 4c). The collagen type III content showed a tendency (*p* = 0.056) to decrease in the ACS group between samples taken on days 0 and 190.

## Discussion

Results of the present study show that a single intralesional ACS injection into SDFT lesions leads to a temporary improvement of ultrasonographic parameters [27, 28]. Transient flattened morphology of tenocyte nuclei and an increased collagen type I expression in ACS-treated tendons over time are indicative of reduced proliferation and increased differentiation of this cell type, respectively [1, 34, 35]. The therapeutic effect of ACS treatment is also demonstrated by an earlier decrease in lameness as compared to controls [2].

The history of the horses was suggestive of strain-induced tendinopathy in the majority of tendons treated with either modality. However, it remains unclear whether the effects shown in the current study equally apply to tendinopathies related to other etiologies. Although the reported duration of tendinopathy of up to 2 weeks before first examination (day 0) referred to clinical signs in the present study, subclinical degeneration is known to precede obvious clinical symptoms of strain-induced tendinopathies, especially in equine athletes [36]. It cannot be excluded that at least some of the tendons on day 0 were not in the inflammatory, but in the early proliferative phase of tendon healing, although neither B-mode ultrasonography nor histology yielded clear evidence of potential chronicity of the tendinopathies. As an alternative, either more extensive tendon biopsies or ultrasonographic tissue characterization as a noninvasive diagnostic tool could have been used initially to further determine the age of the lesions [37, 38].

As tendon composition and biomechanical properties may vary significantly between horses [39] intraindividual controls (control = contralateral limb) are preferred in experimental settings [7]. For these reasons, both front limbs of two horses showing clinical signs of bilateral SDFT tendinopathy were included in the ACS group and as controls. Unfortunately, this could not be realised in more horses. Only two out of five clients who agreed to their horse or the respective limb being included as a control accepted an intralesional injection of these control tendons. Thus, the objective to create two separate control groups, one with the lesion left untreated and one with an intralesional saline injection, could not be achieved. Treatment modalities of control groups in clinical and experimental trials may be seen controversially. On the one hand, it is of interest to compare the effect of controlled exercise plus the effect of the substrate injected intralesionally with the effect of controlled exercise alone (= argument against intralesional injection of a control substance into control tendons). On the other hand, the mere puncture and needle decompression of acute tendon defects may have a therapeutic effect independent of the substrate injected [2]. Against that background, it seems preferable to treat control tendons with sham injections to demonstrate the effect of the substrate injected [5, 7] (ACS in the present case).

Tendon biopsies were used because clinical assessment of tendon healing and B-mode ultrasonography alone are limited with regard to sensitivity and reproducibility [40] and longitudinal needle biopsies were established in human [41, 42] and equine surgery [29, 43] as minimally invasive and well-tolerated techniques. They allow an insight into tendon architecture as well as the immunohistochemical detection of, for example, collagen type I and III [44]. Disadvantages, however, are the potentially therapeutic, albeit unknown, effect of the biopsy process on tendon healing [2, 45], their limited reproducibility and the relatively small volume of tendon tissue harvested [29, 43].

Although mean degree of lameness did not differ between groups, this does not necessarily imply similar functional repair of the lesions, since SDFTs are generally more exposed to maximal load during heavy athletic activities than during trot, i.e. later during rehabilitation [46]. The observation that, compared to day 0, a significant decrease in lameness occurred earlier in the ACS group, i.e. until day 11, compared to the controls (day 36) may be influenced by effects attributed to ACS [8, 11, 13].

Detection of lameness in horses with bilateral SDFT lesions may be more challenging than detection of unilateral gait abnormality [47]. It cannot be excluded that the two horses with bilateral SDFT tendinopathy may have shown bilateral lameness or an additional contralateral lameness, respectively, if diagnostic analgesia had been performed.

Clinical signs of inflammation were monitored using semiquantitative clinical score systems which may be subject to some inaccuracy. This could have been improved by the use of computerized gait analysis, thermography [48] and measurements of the metacarpal circumference in combination with ultrasonography [49]. None of the inflammatory signs, including swelling, differed between groups, but palpable swelling decreased significantly within the ACS group between days 50 and 78 in contrast to controls. On the one hand this may be attributed to auto- and paracrine effects of ACS on endogenous growth factor expression, since in a rodent Achilles tendon transection model bFGF expression was enhanced but not before 8 weeks, i.e. delayed after ACS injection [16]. On the other hand controlled exercise exerted anti-inflammatory effects on tendons from both groups [50] which did not, however, lead to a significant decrease in swelling in controls.

The decrease of palpable swelling in the ACS group between days 50 and 78 correlates positively with the ultrasonographic finding that TL-CSA and %T-lesion decreased only in the ACS group between before treatment and day 78. T-CSA, however, remained unchanged throughout the entire observation period in both groups. This proves that the decrease in swelling in the ACS group rather reflects a decrease in cutaneous, subcutaneous and peritendinous swelling than an altered tendon thickness in the late proliferation and early remodelling phase (i.e. until around day 45). This may be due to a therapeutic effect of inadvertent reflux of small volumes of ACS into the subcutis during intralesional injection. Ultrasonographic measurements of extratendinous swelling are challenging and were not included in the present study, although they could have been helpful to further confirm findings of palpation. In contrast to the results of this study, rat Achilles tendons showed an increase in tendon thickness after ACS treatment compared to the control tendons [22]. However, comparability is limited since the rat tendons, in contrast to the present study, were sutured and received three treatments of ACS.

The present study shows that, compared to control tendons, a single intralesional injection of ACS leads to a significant reduction of %T-lesion and an increase in echogenicity (TES) 78 and 106 days after treatment. This finding corresponds to an earlier (until day 22) increase in TES and an earlier (until day 78) increase of the percentage in parallel orientated fibre bundles (T-FAS) in the ACS group compared to controls. These effects could be the result of stimulation of repair tissue, i.e. improved fibrillogenesis in the early proliferative phase of tendon healing (4–45 days after injury) [23, 28, 37] in which most horses were presented and treated. This may have been a consequence of the potential IL-1Ra-mediated anti-inflammatory action which is attributed to ACS by several authors [8, 11, 13], although IL-1Ra concentrations in ACS were not determined in the present study. Another potential pathway may be the supplementation of growth factors, such as IGF-1 and TGF-β, which are supposed to be increased in equine ACS [12, 16, 21]. IGF-1 is known to be decreased for approximately 2 weeks in experimentally induced tendinopathy and a beneficial bolstering effect of exogenous IGF-1 on low endogenous IGF-1 production during the early repair phase of tendinopathy has been hypothesized [34]. ACS has been shown to display significant effects on the endogenous expression of growth factors potentially via auto- and paracrine pathways [16]. It remains unclear why the significant differences between groups with regard to %T-lesion and TES were not consistent until the end of the observation period despite tendencies to significance. This may be attributed to a time-limited effect of ACS (see above). Ultrasonographic tissue characterization has been established in recent years as a more precise alternative to B-mode ultrasonography to monitor the process of tendon healing [37, 38], particularly if only a probe with a relatively low resolution is available as in the present study.

With regard to histologic scores, a difference between groups was seen at day 36. Here, cell nuclei were flattened in the ACS group compared to controls, which is suggestive of decreased tenocyte proliferation in the late proliferative phase (4–45 days) [1, 23, 34, 35] as a response to the ACS injection. In agreement with this, it has been shown that tendon fibroblasts with a spindle-shaped nucleus have reduced apoptotic and proliferative indices, as demonstrated in human patellar tendons [35]. A consequence might be a decreased cellular production of inelastic collagen type III, which has been described to peak between 3 to 6 weeks after injury in equine experimental studies [34, 51]. However, immunohistochemistry in the present study revealed no difference of collagen type III expression between groups which might be due to considerable variations of different types of collagen between individual lesions, as described for naturally developed lesions in horses [1, 44].

The more favourable development of collagen type I expression in the ACS group between days 36 and 190 indicates qualitative improvement [34, 51], such as increased tensile strength of the repair tissue in the remodelling or maturation phase (45–120 days) [36], which is potentially caused by the mechanisms mentioned, i.e. an IL-1Ra-mediated anti-inflammatory mechanism or the supplementation of growth factors, such as IGF-1 and TGF-β. Collagen type I was seen to be elevated for 6 months after injury in equine experimental studies [34]. This rather reflects the progress in control tendons of the present study and correlates with previous findings in naturally injured equine tendons [44]. In contrast to an experimental Achilles tendinopathy model using ACS-treated rats [22], no difference in collagen type I expression was detected between groups in the present study. However, rat Achilles tendons were treated three times at 24-hour intervals with the first injection 24 hours after induction of the lesion, i.e. in the acute inflammatory phase of tendon healing. By contrast, tendons in the present study received only a single intralesional injection of ACS up to 14 days after the onset of clinical symptoms, i.e. mostly at the end of or even after the acute inflammatory phase. In the latter investigation, real-time quantitative polymerase chain reaction was used, which allows quantification of mRNA transcription of different collagen types, provided that enough tissue is available. Cytokines such as IL-1Ra and the growth factors IGF-1 and TGF-β have a short half-life and they may be degraded and consumed within a short time period after exogenous application [14–16]. Nevertheless, tendon healing may not only be enhanced by direct binding of cytokines and growth factors to cell surface receptors, but also due to indirect effects by stimulation of endogenous production of growth factors [13, 16, 52]. Therefore, the effect of ACS is potentially enhanced by several consecutive injections [22], as reported anecdotally to be common in equine practice and as recommended for the treatment of joint pathology [13]. A single ACS injection was chosen to determine the effect of a low dose as a basis for research because, to date, neither dose-dependent in vivo studies nor a consensus on the treatment protocol are available for blood products such as ACS. Another aim was to keep the number of factors influencing outcome, such as repeated needle puncture of tendons, as low as possible.

The increase in collagen I expression after ACS injection in an experimental rat study did not coincide with an improved maximum load to failure, despite leading to an improvement in tendon stiffness during biomechanical testing [22]. Although biomechanical testing is regarded as the method of choice, it could not be accomplished in the present study due to the inclusion of client-owned horses. In general, the degree of lameness and, to a limited extent, the echo pattern of the injured tendon reflect biomechanical properties. These parameters, however, did not significantly differ between groups in the present study at the end of the observation period. Due to the reduced group size, differences in long-term recurrence rate after return of the horses to full exercise could not be calculated statistically.

## Conclusions

This clinical trial in horses with acute tendinopathies of the SDFT shows that a single intralesional ACS injection contributes to significant reduction of lameness within 10 days and to improvement of ultrasonographic parameters of repair tissue between 11 and 23 weeks after treatment. Intralesional ACS treatment potentially decreases proliferation of tenocytes 5 weeks after treatment and increases their differentiation, as demonstrated by an elevated collagen type I expression in the remodelling phase. Repeated ACS injections should be considered to enhance positive effects. Future controlled long-term investigations should be performed in a larger number of horses to determine the effect on recurrence rate.

## Abbreviations

%T-lesion: Percent total lesion
ACS: Autologous conditioned serum
bFGF: Basic fibroblast growth factor
IGF-1: Insulin-like growth factor-1
IL: Interleukin
IL-1Ra: Interleukin-1 receptor antagonist
MIZ: Maximal injury zone
MSC: Mesenchymal stem cell
SDFT: Superficial digital flexor tendon
T-CSA: Total cross-sectional area
TES: Total echo score
T-FAS: Total fibre alignment score
TGF-β: Transforming growth factor-beta
TL-CSA: Total lesion cross-sectional area
